# Gut microbiota in the pathogenesis and treatment of inflammatory bowel disease: a critical review of mechanisms and therapeutic advances

**DOI:** 10.3389/fmed.2026.1738292

**Published:** 2026-02-24

**Authors:** Yixuan Li

**Affiliations:** Shanghai Jiao Tong University School of Medicine, Shanghai, China

**Keywords:** Crohn's disease, fecal microbiota transplantation, gut microbiota, inflammatory bowel disease, ulcerative colitis

## Abstract

Inflammatory bowel disease (IBD), comprising Crohn's disease (CD) and ulcerative colitis (UC), is a complex, recrudescent chronic gastrointestinal disease. The prevalence of IBD has increased globally year by year, and the exact pathogenesis remains incompletely understood. Evidence indicates that there is a strong correlation between dysbiosis of gut microbiota and the occurrence and progression of IBD. This review systematically describes recent advances in understanding the role of gut microbiota in IBD, with a particular focus on how dysbiosis contributes to pathogenesis. In addition, this review synthesizes the latest research progress and challenges of therapies of IBD targeting the gut microbiota, highlighting both their therapeutic potential and current limitations. Importantly, literature is based on targeted selection of high-quality sources, including clinical trials, meta-analyses, systematic reviews, and regulatory documents, to provide a balanced and up-to-date perspective. Emphasis is laid on the potential of microbiota-targeted therapies in IBD management.

## 1 Introduction

Inflammatory bowel disease (IBD), which consists of Crohn's disease (CD) and ulcerative colitis (UC), is a complex and chronic condition with inflammation of the gastrointestinal tract. The prevalence of IBD increased substantially in many regions from 1990 to 2017, threatening a heavy social and economic burden on governments and health systems in the future (1). Adolescents and young adults aged 15–39 are the age groups most susceptible to IBD, which constitute a significant proportion of its disease burden and are expected to persist through 2040 (2). Gastrointestinal symptoms of IBD include abdominal pain, diarrhea, hematochezia, and vomiting, resulting in malnutrition and weight loss. In addition, psychiatric disorders caused by IBD such as anxiety and depression often coincide with a decrease in the patients' quality of life (3). A combination of complicated factors such as genetic background, environment, host immune response are associated with IBD. Gut microbiota comprises bacteria, viruses, protozoa and fungi, among which bacteria are the most abundant. Bacteria in the gastrointestinal tract play a fundamental role in nutrition, immune, metabolism, and defense against pathogens in the host (4). In recent years, dysbiosis of gut microbiota has been widely viewed as an essential factor in the pathogenesis of IBD. Therefore, exploring the influence of gut microbiota in IBD is of vital importance, which provides potential targets for the treatment of the disease. This review synthesizes recently emerged key mechanistic studies, and elaborates their precise roles in intestinal barrier dysfunction and immune homeostasis disruption. Furthermore, it integrates the latest advances and challenges in microbiota-related therapeutic strategies, while critically discussing their efficacy heterogeneity, safety concerns, and challenges for personalized application. The aim of the article is to provide researchers and clinicians with a balanced perspective that bridges mechanistic insights with translational applications, thereby advancing the development of personalized management strategies for IBD.

## 2 Pathophysiology

### 2.1 Dysbiosis of gut microbiota in IBD

In healthy individuals, gut microbiota maintain diversity and preserve the advantage of beneficial bacteria (such as those producing short-chain fatty acids, SCFAs), which contributes to homeostasis of the gut environment. By contrast, in IBD patients, the species and distribution of gut microbiota are significantly altered, indicating a state of dysbiosis (5). Studies aimed at the gut microbiota in IBD patients indicate a generalized decrease in biodiversity (6). Studies have identified an increase in Proteobacteria and Bacteroidetes and a decrease in Firmicutes in CD patients (7). In addition, the reduction in beneficial SCFA-producing bacteria, such as *Bifidobacterium longum, Eubacterium rectale, Faecalibacterium prausnitzii*, and *Roseburia intestinalis*, has been shown in UC patients (8). These results illustrate that dysbiosis in IBD not only manifests as changes in the number of microbial strains, but also involves an overall dysfunction of gut microbiota. Furthermore, besides bacterial dysbiosis, fungal microbiota dysbiosis is also observed in IBD, with an increased Basidiomycota/Ascomycota ratio, a decreased proportion of *Saccharomyces cerevisiae* and an increased proportion of *Candida albicans* (9). Among these changes in gut microbiota, the reduction of SCFA-producing bacteria is considered most crucial and translationally promising, in view of its central role in mucosal immunity and energy metabolism.

### 2.2 Interactions between gut microbiota and intestinal mucosal barrier

The intestinal mucosal barrier is composed of mechanical barrier, chemical barrier, immune barrier and biological barrier. The complete intestinal mucosal epithelial cells and intercellular tight junctions form the structural basis of mechanical barrier of the intestine, effectively protecting the host from potentially harmful substances. The gut microbiota has been identified as a key factor in maintaining intestinal mucosal barrier microenvironment homeostasis. Recent studies have shown that colonization with *Akkermansia muciniphila* stimulates the expression of the *NLRP6* inflammasome and activates autophagy in goblet cells which produce new mucins, protecting the intestinal mucosal barrier (10). In addition, studies have demonstrated that *Lactobacillus paracase* E10 can increase goblet cell density and upregulate tight junction proteins, thereby enhancing intestinal barrier integrity (11). These studies consistently indicate the direct effects of specific microbial strains in maintaining the integrity of the intestinal mucosal barrier. By contrast, the enterotoxins produced by pathogenic bacteria including Enterobacteriaceae and Bacteroidaceae can severely damage the epithelial structure of the host and induce increased intestinal permeability. Decrease of beneficial bacteria and increase of potentially harmful bacteria lead to mucus abnormalities, which is likely to contribute to IBD pathogenesis.

### 2.3 Effects of the gut microbiota bacteria metabolites

Gut microbiota can produce SCFAs during the fermentation of dietary fiber or non-digestible carbohydrates, which play a role in regulating intestinal pH, promoting mucus production, providing energy for epithelial cells, and enhancing mucosal immunity. Acetate, propionate, and butyrate are the three most abundant SCFAs derived from gut microbiota. Microbiota-derived SCFAs have been confirmed to be able to effectively mitigate *TNF-*α-induced oxidative stress by modulating antioxidant enzyme activity, alleviating the inflammatory process in IBD (12). Decrease in SCFAs production affects the energy supply and immune regulation of intestinal cells. A recent meta-analysis has demonstrated a significant reduction in fecal SCFAs levels in patients with IBD, particularly during active phases of the disease and most markedly in CD (13). These studies indicate that the reduction of SCFAs is an essential metabolic marker of IBD and plays a crucial role in the pathogenesis and development of the disease.

Indole-3-acetic acid (IAA) is another gut microbiota metabolite produced by intestinal bacteria such as *Lactobacillus reuteri*, which enhances intestinal mucin sulfation through the *AHR-Papss2-Slc35b3* pathway, contributing to the protection of intestinal homeostasis. When tryptophan metabolism in the gut microbiota is disrupted by factors such as high-fat diet, lower levels of IAA lead to disease aggravation in both IBD patients and murine colitis models (14). In addition, indole-3-propionic acid (IPA) together with the branched-chain acids (BCFAs) and the SCFAs produced by *Clostridium sporogenes* protect mice against colonic inflammation by up-regulating *IL-22* gene expression (15). These research results are consistent in the pivotal role of gut microbiota-derived metabolites in the maintenance of intestinal homeostasis, which provide a potential target for IBD therapies.

### 2.4 Interactions between gut microbiota and immune system

Gut microbiota plays a pivotal role in immune regulation, which is an essential factor in the pathogenesis of IBD. Beneficial bacteria in the gut microbiota can play an immunosuppressive role by regulating host immune cells, while harmful bacteria can induce inflammatory cytokines by immune cell interactions (16). Through complex interactions with immune cells, gut microbiota modulates both innate and adaptive immune responses. This section highlights the interactions between gut microbiota and CD4+T cells, macrophages, innate lymphoid cells, and neutrophils, and discusses how these interactions contribute to the pathogenesis of IBD.

**Figure 1:**
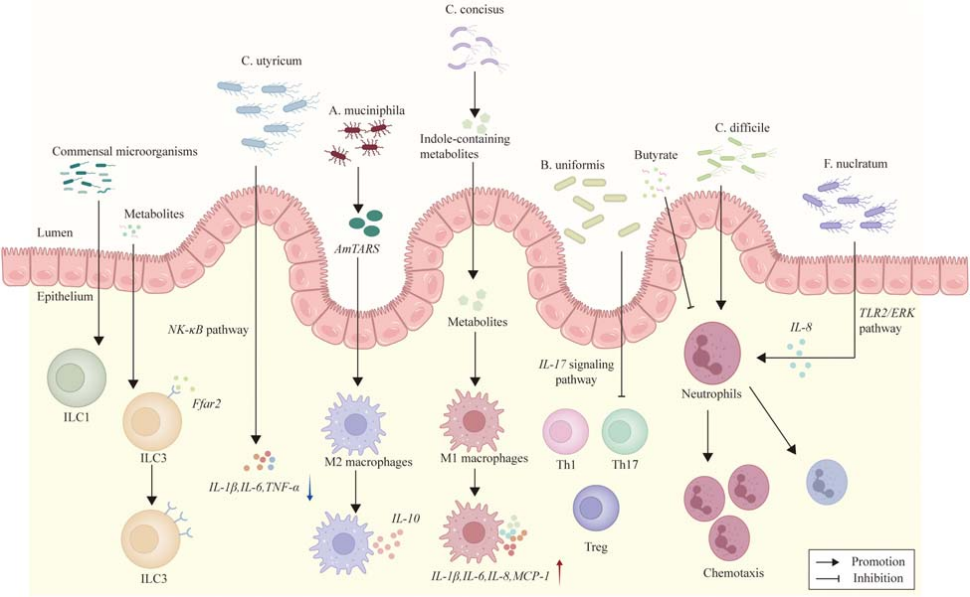
The figure illustrates interactions between certain gut microbiota and immune cells. *B. uniformis* inhibits IL-17 signaling pathway activity and Th17 cell differentiation. *C. butyricum* diminishes pro-inflammatory cytokines by downregulating NF-κB signaling pathway. *A. muciniphila* secretes AmTARS, which triggers M2 macrophage polarization and orchestrates the production of IL-10. *Campylobacter concisus* produces indole-containing metabolites which induce the release of pro-inflammatory cytokines IL-1β, IL-6, IL-8, and MCP-1. The development of ILC1s is dependent on commensal microorganisms. ILC3s express Ffar2, a microbial metabolite-sensing receptor whose agonism promotes ILC3 expansion and function. Butyrate suppresses neutrophil-associated immune responses. *C. difficile* promotes neutrophil recruitment. *F. nucleatum* induces neutrophil chemotaxis, which depends on IEC-derived IL-8 secretion and mediated by the TLR2/ERK signaling pathway.

The interactions between gut microbiota and key components of immune system are presented in Figure 1.

#### 2.4.1 Interactions with CD4+T cells

CD4+T cells are crucial factors in IBD development. CD4+T cells can differentiate toward a range of distinct phenotypes such as T helper(h)1, Th17, and several types of T-regulatory cells (Tregs). Increased Th1/Th17 cell differentiation and reduced Tregs differentiation result in enhanced susceptibility to IBD, due to increased production of pro-inflammatory cytokines (*IL-6, IL-1*β, *IL-17*, and *TNF-*α) and decreased expression of immune regulatory factors (*IL-10*, retinoic acid, and IDO) (17). A study has demonstrated that *Bacteroides uniformis* alleviates colitis by inhibiting *IL-17* signaling pathway activity and Th17 cell differentiation (18). Another study has shown that *C. butyricum* diminishes pro-inflammatory cytokines like *IL-1*β, *IL-6* and TNF-α by downregulating nuclear factor kappa-B (*NF-*κ*B*) signaling pathway (19). Compelling evidence demonstrates that specific bacteria can directly skew the Th17/Treg balance by modulating certain signaling pathways, which aligns with the pivotal role of T cell dysregulation in the pathogenesis of IBD.

#### 2.4.2 Interactions with macrophages

Macrophages are essential for the development of IBD, among which M1 and M2 play critical roles in the damage and repair of intestinal homeostasis. M1 macrophages can secrete pro-inflammatory cytokines such as *IL-1*β, *IL-6, IL-12*α, *IL-23*, and *TNF-*α, while M2 macrophages can secrete anti-inflammatory cytokines *IL-10*. *A. muciniphila* can secrete the threonyl-tRNA synthetase (*AmTARS*), which triggers M2 macrophage polarization and orchestrates the production of *IL-10* (20). *Campylobacter concisus* can produce indole-containing metabolites which induce the release of pro-inflammatory cytokines *IL-1*β, *IL-6, IL-8*, and *MCP-1* and increase the recruitment and activation of macrophages (21). These studies indicate the key role of gut microbiota in the polarization and activation of macrophages. The opposing effects in immune regulation of the above two bacteria illustrate that the microbial influence on immunity is strain-specific. Overall, targeting macrophage polarization remains a major therapeutic frontier, in which using certain microbial products to drive M2 macrophage polarization is especially a highly precise and promising approach.

#### 2.4.3 Interactions with innate lymphoid cells (ILC)

ILCs are mainly located in mucosal tissues, which are essential in the maintenance of barrier functions and in the initiation of an appropriate immune response upon pathogenic infection. The family of ILCs are classified into three groups: ILC1s producing *IFN-*γ, ILC2s producing *IL-5* and *IL-13*, and ILC3s producing *IL-22* and/or IL-17 (22).

Signals from commensal gut bacteria can promote good expression of cytokines in the intestine and significantly affect the function of ILCs. The development of ILC1s is dependent on commensal microorganisms, while the development of ILC2s does not require microbiota (23).

Colonic ILC3s are found to express *Ffar2*, a microbial metabolite-sensing receptor, whose agonism promotes ILC3 expansion and function. Deletion of *Ffar2* in ILC3s leads to impaired gut epithelial function, which is associated with the development of IBD (24). In another study, glucagon-like peptide-1 receptor agonists (GLP-1RAs) enhance ILC3s cytokines-producing to alleviate DSS-induced colitis, and the regulatory effect requires the involvement of the gut microbiota (25). These results demonstrate the close interactions between gut microbiota and ILCs. Emphasis is laid on the crucial role of gut microbiota metabolites and their receptors in the precise regulation of ILC3s functions and intestinal immunity. However, more specific molecular mechanisms need to be identified.

#### 2.4.4 Interactions with neutrophils

Neutrophils are implicated in the development of IBD as first responder of immune cells. Neutrophils in large numbers infiltrate in the inflamed mucosa and accumulate in the epithelia, causing damage of mucosal architecture and releasing neutrophil extracellular traps (NETs) (26).

Li et al. (27) investigated how microbiota-derived metabolites such as butyrate regulate functions of neutrophils in the pathogenesis of IBD. *In vitro* studies confirmed that butyrate suppressed neutrophil migration and formation of NETs from both CD and UC patients (27). Consistently, in murine studies, oral administration of butyrate markedly ameliorated mucosal inflammation in DSS-induced colitis through inhibition of neutrophil-associated immune responses (27). Dong et al. reported that *Clostridioides difficile* aggravated DSS-induced colitis by shaping the gut microbiota and promoting neutrophil recruitment. The isolated neutrophils from *C. difficile*-infected mice with colitis showed a robust migratory ability and had enhanced expression of cytokines and chemokines (28). Another study reported that Fusobacterium nucleatum induced neutrophil chemotaxis and decreased the integrity of intestinal epithelial cells (IEC), which was dependent on IEC-derived *IL-8* secretion and mediated by the *TLR2/ERK* signaling pathway (29). These preclinical studies illustrate bidirectional regulation of microbial factors on neutrophil. Microbial metabolites like butyrate can suppress neutrophil recruitment and NET, while certain microbiota can exacerbate inflammation via neutrophil chemotaxis, which may both serve as novel therapeutic potential in the treatment of IBD.

The above results exemplify the effect of gut microbiota in modulating host immune pathways, reinforcing the close association between gut microbiota and immune system. Among these mechanisms, the interactions with CD4+T cells and macrophages represent highly critical and immediately targetable pathways, as they play a pivotal role in the immune dysregulation in IBD. Meanwhile, the microbial metabolite-sensing receptor of ILC3 remains a rather promising mechanistic insight for future precise therapies. As for neutrophil interactions, their modulation may perform broader anti-inflammatory functions, rather than core modifying effects on the pathogenesis of IBD.

### 2.5 The causal relationship between dysbiosis and IBD pathogenesis

Whether the dysbiosis of gut microbiota in IBD is a primary causative driver of inflammatory process or a secondary consequence of the dysregulated intestinal mucosal barrier and immune system remains a core and unresolved question. The above mechanistic studies demonstrated microbial factors' interactions with intestinal mucosal barrier and immune system, which supports the case for causality. However, significant challenges complicate causal inference, including genetic factors, individual diet, and medications especially antibiotics. Furthermore, inflammation itself can provide terminal electron acceptors for anaerobic respiration and can support blooms of facultative anaerobes, such as *Escherichia coli*, in inflamed gastrointestinal tracts (30), which indicates dysbiosis could be a secondary consequence of inflammation. Longitudinal cohort studies are prioritized for distinguishing causative microbial factors from the complex dysbiosis of IBD.

## 3 Emerging IBD therapies targeted at gut microbiota

Gut microbiota-targeted treatments have emerged as a promising therapeutic strategy, in view of the crucial role of gut microbiota in the pathogenesis of IBD. This section lays emphasis on the latest advances and clinical evidence of gut microbiota-targeted treatments including probiotics, prebiotics, dietary interventions and fecal microbiota transplantation. Besides, other interventions targeted at gut microbiota include symbiotic therapy, postbiotic therapy and phage therapy (31, 32).

### 3.1 Probiotics

Probiotics are living microorganisms that can regulate intestinal microflora, enhance intestinal mucosal barrier and exert anti-inflammatory effect, thereby alleviating the symptoms of IBD patients (33). In recent years, probiotics have attracted increasing attention as a new type of therapeutic intervention. However, the efficacy of probiotics in IBD remains a controversial subject, with clinical trials yielding conflicting results (34–40). This heterogeneity may stem from differences in probiotic strains, formulations, patient populations and study endpoints.

There is not obvious evidence of side effects of probiotic interventions in IBD. Nevertheless, microorganisms used as probiotics may cause systemic infections, stimulate the immune system, disturb metabolism and participate in horizontal gene transfer (41).

The probiotic studies on IBD in recent years are presented in Table 1.

**Table 1:** Recent probiotic studies on IBD.

**References**	**Study design**	**Subject**	**Probiotic intervention**	**Key findings**
Bamola et al. (34)	RCT, double blind	IBD patients	*B. clausii* UBBC-07 vs. placebo	Modulated gut microbiota and cytokines. Alleviated inflammation.
Fennessy et al. (35)	RCT, double blind	IBD patients with IBS	Four-strain probiotic vs. placebo	No difference in IBD outcomes. More significant reduction in IBS symptoms.
Shen et al. (36)	RCT	CD patients	Mesalamine with live combined probiotics vs. mesalamine only	Clinical efficacy. Improved composition of gut microbiota. Increase in anti-inflammatory cytokines.
Hoteit et al. (37)	RCT, single blind	CD patients	*Lactobacilli, Bifidobacteria*, and *Lactococcus bacillus* vs. Placebo	No significant alterations in symptoms. Improved body composition, nutrient intake, and QoL.
Prosberg et al. (38)	RCT, double blind	UC patients	*Trichuris suis* ova vs. placebo	No clinical or endoscopic remission at week 24. Symptomatic temporary remission at Week 12.
Agraib et al. (39)	RCT, double blind	UC patients	Capsules containing nine *Lactobacillus* and five *Bifidobacterium* species vs. placebo	Induced remission. Reduction in immunoglobulins and inflammatory markers. Increased IL-10 levels.
Park et al. (40)	RCT, double blind	UC patients	*E. coli* Nissle 1917 vs. placebo	Additive clinical response at 4 weeks and endoscopic remission at 8 weeks.

RCT, randomized controlled trial; IBS, irritable bowel syndrome; QoL, quality of life.

Overall, current evidence demonstrates the inconsistent efficacy of probiotics on alleviating the core symptoms of IBD, which may be caused by specificity of probiotic strains, differences in disease phenotypes, and variety of individuals. Moreover, most of the above probiotic trials are underpowered, short-term, not rigorously blinded, and prone to placebo effects. Neglect of the host's baseline microbiome and immune status remains a significant limitation, which leads to the inconsistent therapeutic efficacy of probiotics on different IBD patients. Nevertheless, probiotics have shown certain adjunctive therapeutic potential in immune regulation, microbiota modulation, nutrition enhancement, and improvement of quality of life.

### 3.2 Prebiotics

Prebiotic refers to a substrate that is selectively utilized by host microorganisms conferring a health benefit (42). Sources of prebiotics include breast milk, soybeans and raw oats, while the most popular prebiotics are the oligosaccharides contained in plants (43). As an alternative or supplement to probiotics, prebiotics promote probiotics growth, selectively stimulate intestinal microorganisms such as *Bifidobacterium* and *Lactobacillus* strains, influence the production of SCFAs and help preserve intestinal barrier integrity (44), which is viewed as a novel intervention in IBD.

Inulin, a water-soluble storage polysaccharide, is a non-digestible carbohydrate rich in chicory root (45). Cao et al. (46) reported that a colon enzyme-activated prebiotic nanomedicine containing inulin can promote a beneficial shift in gut microbiota by decreasing pathogenic bacteria and increasing beneficial bacterial populations. Similarly, Wu et al. (47) developed an inulin hydrogel loaded with self-assembled nanoparticles of curcumin and glycyrrhizic acid for IBD treatment, which restored intestinal microbial homeostasis and ameliorated disease activity index in DSS-induced colitis mice. Another study on inulin also observed changes in gut microbiota composition, with an increase in SCFAs and a reduction in pro-inflammatory cytokine expression (48).

Furthermore, recent studies have laid emphasis on prebiotics from different sources (49–51). A study demonstrated the colitis-alleviating properties of *Lactiplantibacillus plantarum* dy-1 fermented barley (LFBE), which contains the prebiotic barley matrix. In further gut microbiota analysis, the proliferation of *Akkermansia* and *Lachnospiraceae* was promoted, while the colonization of *Escherichia-Shigella* was inhibited (49). A clinical trial on CD patients reported that mushroom based prebiotic supplement resulted in increase in SCFA-producing bacteria, such as *Parabacteroides distasonis* and *Faecalimonas umbilicata* (50). A study on mice with DSS-induced colitis investigated an oligomeric procyanidins-enriched grape seed extract. Compared to a standard diet, the prebiotic increased beneficial bacteria and decreased potentially harmful bacteria, which markedly protected mice against DSS effects (51).

These studies illustrate that specific prebiotics promote beneficial bacteria growth and enhance intestinal barrier function. However, most clinical trials fail to mechanistically associate prebiotics administration with a sustained microbial shift and a definitive clinical outcome. Furthermore, the prebiotics trials lack dose-response standardization, and may cause intolerance reactions at effective doses, which limit their application as standalone therapies of IBD.

### 3.3 Dietary interventions

Diet is viewed as an important modifiable factor in the pathogenesis and treatment of IBD, which can remarkably improve both the composition and functionality of the gut microbiota.

#### 3.3.1 Crohn's disease exclusion diet (CDED)

CDED is an exclusion diet scheme featuring in eliminating ingredients that may contain potential triggering factors for CD, which is often used alongside partial enteral nutrition (PEN) (52). A trial on adults with mild-to-moderate CD demonstrated that both CDED plus PEN and CDED alone were effective for induction and maintenance of remission and might lead to endoscopic remission (53). Levine et al. (54) reported that CDED plus PEN produced changes in the fecal microbiome and induced sustained remission in a significantly higher proportion of patients than exclusive enteral nutrition (EEN). Verburgt et al. investigated the effect of CDED plus PEN on correcting compositional or functional dysbiosis in 54 pediatric CD patients. A significant increase in Firmicutes, particularly Clostridiales, and a significant decrease in Proteobacteria, particularly Gammaproteobacteria were observed. However, 12 weeks of diet was not enough to achieve complete correction of dysbiosis (55). Though these above results indicate the significant therapeutic effect of CDED by modulating gut microbiota, the individual adaptability and adherence to the dietary plans remain severe challenges in clinical implementation of the treatment.

#### 3.3.2 Ketogenic diet (KD)

Ketogenic diet (KD) is a dietary therapy characterized by high-fat and low-carbohydrate, which mediates the rise of circulating ketone bodies and exerts a potential anti-inflammatory effect (56). Kong et al. investigated the effect of KD on gut microbiota. Compared with low-carbohydrate diet (LCD), KD led to a reproducible increase in *Akkermansia* and a decrease in *Escherichia*/*Shigella* (57). A case report also confirmed clinical improvement and higher quality of life in IBD patients with KD (58). However, another study demonstrated the opposite result that KD substantially worsened colitis in mice and led to increase in pathogenic taxa such as Proteobacteria, Enterobacteriaceae, Helicobacter and *Escherichia-Shigella*, and decrease in potential beneficial taxa such as *Erysipelotrichaceae* (59). Inconsistency of results may indicate the effect of variation in species, individuals, dietary formulas, and intervention durations. Further research on larger scale should be conducted.

#### 3.3.3 Mediterranean diet (MD)

The Mediterranean diet (MD) is characterized by a high consumption of vegetables, fruits, cereals, nuts, legumes, unsaturated fat such as olive oil, a medium intake of fish, dairy products, wine, and a low consumption of saturated fat, meat, and sweets (60), which has been confirmed to be associated with the reduction of inflammatory markers (61). Evidences suggest that MD is able to modulate the gut microbiota and increase its diversity.

A study reported that compared with a Western food model, the microbiota in MD would produce more SCFAs (62). This result corresponds with another study conducted by Muralidharan et al. The study suggested the role of MD changes on the host might be via SCFA producing bacteria, with a decrease in *Butyricicoccus, Haemophilus, Ruminiclostridium 5*, and *Eubacterium hallii* (63). In addition, olive oil, a crucial component of MD, has been reported to provide a considerable anti-inflammatory effect in IBD. A study indicated that the consumption of extra virgin olive oil was related to the increase of the genus Clostridium XIVa responsible for SCFA production (64). In another study, *L. plantarum* SC-5 combined with olive oil extract tyrosol demonstrated a decrease in Proteus and an increase in *Lactobacillus, Bifidobacterium*, and *Akkermansia*, thereby ameliorating the colonic inflammation in mice (65). These results highlight MD as a promising dietary intervention which induces IBD remission in a gut microbiota-dependent manner.

#### 3.3.4 Other dietary patterns

Furthermore, several studies (66–68) on diversified dietary patterns have been conducted, providing potentially innovative strategies for IBD management. A study concentrated on the long-term soy dietary fiber diet. On chronic UC mice, high-purity soy isolate dietary fiber played an important role in the maintenance of intestinal bacterial flora by inhibiting the degradation of *L. intestinalis* and promoting the proliferation of Oscillospira, which provided novel ideas for the treatment of the disease (66). Ye et al. investigated the effects of a reduced sulfur (RS) diet on the gut microbiome (GM) composition in individuals with remitted or active UC. The RS diet increased beneficial microbes while decreasing pathobionts, thereby reducing inflammation (67). Another study reported dietary interventions with oats or oat bran modulated the composition of gut microbiota and increased the content of SCFAs (68). These studies elucidate the ability of specific diet pattern to alter microbial communities and ameliorate inflammatory outcomes, which highlights dietary intervention as an effective and modifiable approach in IBD treatment.

Though microbiota-targeting dietary therapies like CDED, KD and MD demonstrate considerable promise in IBD remission, severe challenge lies in long-term adherence and practicality, which impacts their real-world implementation. Most clinical trials merely show short-term efficacy in modulating gut microbiota and ameliorating inflammation, and placebo effects are inevitable. Additionally, dietary responses tend to be influenced by individual baseline microbiome along with cultural and socioeconomic factors, which pose a challenge to establishing universal protocols.

### 3.4 Fecal microbiota transplantation (FMT)

FMT involves transplanting the functional microbiota from healthy donor feces into the patient's gastrointestinal tract to rebuild a new GM. FMT modulates gut bacteria abundance and restores microbial diversity, which has emerged as a promising therapeutic approach for IBD. Nevertheless, the exact mechanisms of action are not yet fully understood (69). A study suggested FMT suppressed the epithelial-mesenchymal transition and *Wnt/*β*-catenin* pathways (70), yet the precise mechanism remains unclear.

#### 3.4.1 FMT in CD treatment

Excessive colonic bile acids (BA) influx in IBD results in bile acid malabsorption (BAM) and bile acid diarrhea (BAD), affecting about 30% of CD patients with ileal involvement (71).

A clinical trial found that IBD patients especially CD patients with BAM were intended to have more obvious improvement in clinical response (66.67%) and remission (52.38%) after FMT treatment, with alleviation in abdominal pain and diarrhea (72).

Exclusive enteral nutrition (EEN) is an effective therapy for remission induction in pediatric CD, with a disadvantage of high relapse rates after return to a regular diet (73). Hoelz et al. tested the feasibility of autologous FMT after EEN in pediatric CD patients. Insufficient amount of fecal material, inadequate stool consistency and low microbial diversity indicated that autologous FMT via capsules containing EEN-conditioned microbiota was unlikely to be a suitable therapeutic approach in pediatric CD (74).

Data on FMT for CD are more limited than UC. With mixed results in published literatures, currently, FMT has not been viewed as a standard therapy for CD. More researches should be conducted to assess FMT's efficacy in CD treatment.

#### 3.4.2 FMT in UC treatment

FMT has been confirmed by several studies to be effective in relieving or curing active UC (75). Crothers et al. conducted a study on daily, oral FMT for long-term maintenance therapy in UC. Two subjects that received encapsulated oral FMT (cFMT) achieved clinical remission vs. none in the placebo group, which was associated with sustained donor-induced shifts in fecal microbial composition (76). To identify the long-term impacts of cFMT on the colonic microbiome, larger trials should be done. Raich et al. investigated bacterial taxonomic and functional changes following oral lyophilized donor FMT in patients with UC. The GM of patients treated with oral lyophilized FMT significantly increased in species-genome bin richness compared with patients receiving placebo (77).

However, a study identified that when a low richness GM was transferred to a recipient with a high richness GM, the donor GM failed to successfully colonize, which exacerbated DSS-induced colitis (78). This effect may partly explain why there are instances in which FMT has been ineffective or even exacerbated IBD in patients. Meanwhile, this result illustrates that the success of FMT highly depends on compatibility of donor and recipient microbiota and the efficiency of colonization after transplantation. In addition, low richness of donor GM is a crucial risk factor causing failure of transplantation and aggravation of disease.

#### 3.4.3 Adverse events in FMT

The adverse events in FMT remain an essential challenge. In a systematic review, the total incidence rate of adverse events in FMT was 28.5% (79), most of which were mild or moderate and self-limiting. A meta-analysis reported serious adverse events related to FMT developed in less than 1% of patients, including sepsis, aspiration pneumonia, and bowel perforation (80), which underscore the importance of rigorous donor screening and standardized administration. Most FMT-related deaths were reported in patients receiving FMT via the upper gastrointestinal route, and all reported FMT-related serious adverse events were in patients with mucosal barrier injury (81). To bring FMT into mainstream clinical practice, standardized procedures, careful donor screening to reduce risks, and strategies to handle the rare but serious complications that can arise are in urgent need.

FMT represents a potent and complicated microbiota-targeting intervention. Its efficacy in IBD remission highly depends on donor-recipient compatibility and successful engraftment, which are still unpredictable and uncontrollable factors. Serious safety risks and the lack of standardized administration remain major hurdles to FMT's real clinical implementation. Fully understood mechanisms, predictable positive and negative effects, and established safety protocols are indispensable for FMT to evolve into a mature clinical regimen.

## 4 Conclusions

This review demonstrates that dysbiosis of gut microbiota plays an essential role in the pathogenesis of IBD. By weakening the intestinal mucosal barrier and disrupting the immune system, gut microbiota disturbs intestinal homeostasis and drives chronic inflammation. However, the causality between dysbiosis and IBD has not been elucidated yet, which is currently the foremost limitation in mechanistic studies and clinical translation. It remains uncertain whether dysbiosis is a primary driver or a secondary consequence of IBD. Though gut microbiota have been reported to involve in *FXR/TGR5/PXR* pathway, *AhR-IL-22* pathway, and *TLR4/NOD2/NF-*κ*B* pathway (82), the exact mechanism needs further studies to clarify.

This review highlights several key therapeutic strategies targeting gut microbiota, including probiotics, prebiotics, dietary interventions and FMT, which have emerged as novel paradigms for treatment. A large amount of evidence has indicated the efficacy of these treatments in clinical remission and improvement of quality of life. However, there are still significant challenges such as side effects of treatments, variable response among individuals, unclear mechanisms of action, and lack of standardized protocols. The lack of effective biomarkers remains a crucial limitation in the assessment of therapeutic efficacy. Several serological biomarkers, miRNAs and potential genes such as IRF1 are expected to be novel biomarkers of IBD (83, 84). In addition, most clinical trials are limited by small sample sizes, short follow-up periods, heterogeneous study designs and a lack of analysis with concomitant treatment.

Foresightedly, identifying causative microbial drivers from the overall dysbiosis in IBD is of highest priority, which enables the precise selection of disease-alleviating gut strains in the therapies. Conducting rigorous and well-designed clinical trials is also in relatively urgent need for durable and definitive treatment effects. Future mechanistic researches should prioritize longitudinal cohort studies, and focus on elucidating the exact molecular pathways in the interactions between gut microbiota and host. Future clinical research should prioritize trails with larger scale and longer follow-up to assess the efficacy of the treatment. Safety of the microbiota-targeted treatments should be evaluated and confirmed. Personalized therapy that considers individual microbiome characteristics, immune system profile and dietary habits may improve the specificity of treatment. Furthermore, the integration of microbial engineering, synthetic ecology, multi-omics technologies and artificial intelligence will deepen understanding of host-microbiota interactions and ultimately enable effective and safe microbiota-targeted therapies for each IBD patient.
